# Estrogen Acts Through Estrogen Receptor-β to Promote Mannan-Induced Psoriasis-Like Skin Inflammation

**DOI:** 10.3389/fimmu.2022.818173

**Published:** 2022-05-19

**Authors:** Huimei Wu, Longhui Zeng, Jiaxin Ou, Tingting Wang, Yong Chen, Kutty Selva Nandakumar

**Affiliations:** ^1^ Southern Medical University - Karolinska Institute United Medical Inflammation Center, School of Pharmaceutical Sciences, Southern Medical University, Guangzhou, China; ^2^ Department of Orthopedics, Guangdong Provincial People’s Hospital, Guangdong Academy of Medical Sciences, Guangzhou, China; ^3^ Department of Rheumatology and Immunology, Shenzhen People’s Hospital, The Second Clinical Medical College of Jinan University, The First Affiliated Hospital of Southern University of Science and Technology, Shenzhen, China

**Keywords:** psoriasis, estrogen, estrogen receptor β, γδ T cells, dendritic cells, IL-23/IL-17 axis

## Abstract

Sex-bias is more obvious in several autoimmune disorders, but not in psoriasis. However, estrogen levels fluctuate during puberty, menstrual cycle, pregnancy, and menopause, which are related to variations in psoriasis symptoms observed in female patients. Estrogen has disease promoting or ameliorating functions based on the type of immune responses and tissues involved. To investigate the effects of estrogen on psoriasis, at first, we developed an innate immunity dependent mannan-induced psoriasis model, which showed a clear female preponderance in disease severity in several mouse strains. Next, we investigated the effects of endogenous and exogenous estrogen using ovariectomy and sham operated mice. 17-β-estradiol (E2) alone promoted the skin inflammation and it also significantly enhanced mannan-induced skin inflammation. We also observed a prominent estrogen receptor-β (ER-β) expression in the skin samples, especially on keratinocytes. Subsequently, we confirmed the effects of E2 on psoriasis using ER-β antagonist (PHTPP) and agonist (DPN). In addition, estrogen was found to affect the expression of certain genes (*vgll3* and *cebpb*), microRNAs (miR146a and miR21), and immune cells (DCs and γδ T cells) as well as chemokines (CCL5 and CXCL10) and cytokines (TNF-α, IL-6, IL-22, IL-23, and IL-17 family), which promoted the skin inflammation. Thus, we demonstrate a pathogenic role for 17-β-estradiol in promoting skin inflammation, which should be considered while designing new treatment strategies for psoriasis patients.

## Introduction

The immune system in men and women differs significantly, especially after puberty (1) and a conservation in sex bias during expression of genes promotes phenotypic differences (2). Women have two X chromosomes containing many immune response related genes expressing Toll-like receptors, cytokine receptors, and contributing to T and B-cell immunity as well as their regulation (3). Thus, women are comparatively better than men in having good health, longevity, and a stronger immune response to infections but this immunological advantage might contribute to susceptibility to autoimmune diseases (4). It is well known that the autoimmune diseases have gender bias, with female preponderance. During pregnancy, systemic lupus erythematosus worsens, while multiple sclerosis, rheumatoid arthritis, autoimmune thyroid diseases, and others improve (5) but psoriasis development in pregnant women is controversial. Therefore, how sex hormones affect the immune system and autoimmune disorders needs more experimental investigations.

Psoriasis affects about 25 million people in North America and Europe (6) in which genetic (7) and environmental factors (sex, emotional stress, smoking, etc.) are involved (8). The ratio of psoriasis incidence between women and men was reported to be 1.05 (9). However, the intensity and onset of psoriasis in men and women are different (10). Women undergo sweeping endocrinological changes in their lifetime (puberty, menstrual cycles, pregnancy, and menopause periods) and, show great variations in psoriasis development (11). During pregnancy, 55% of psoriasis patients had improvement, while 23% of them had an aggravated disease (12). Another study reported amelioration of psoriasis during pregnancy, though it relapsed in the puerperium period. Interestingly, psoriasis arthritis became highly severe in these patients after delivery (13). Of note, approximately 30% of Ps patients develop PsA and a strong association of different genes was reported for PsA and Ps (14). Women with irregular menstrual cycles had a higher psoriasis incidence with a ratio of 1.32 when 163,763 people were used in a study protocol (15).

Estrogen is one of the factors contributing to sex differences, which directly affects keratinocyte proliferation, IL-17-producing γδ T cells (16), and infiltration of macrophages (17) and dendritic cells (18). It also increases IL-23/IL-23R signaling and IL-17A production from Th17 cells (19). In psoriasis, estrogen might exert its functions by modulating the expression of certain genes like sex-biased transcription co-factor vestigial-like protein 3 (*Vgll3*) (20) and CCAAT enhancer binding protein beta (*cebpb*) (21), microRNAs (miR146a, 21 and 210) (22, 23), and chemokines (CCL5 and CXCL10) (24, 25). Estrogen mainly binds to nuclear receptors (ER-α and β) to mediate its functions but contribution of each receptor varies depending on the target tissue and disease conditions (26, 27). At the same time, estrogen could have disease promoting or ameliorating functions based on the type of induced immune responses and the target tissues (26, 27). Symptoms of psoriasis and psoriatic arthritis were reported to develop in reactive oxygen species (ROS)-deficient B10Q.Ncf1^m1j/m1j^ mice after an intraperitoneal injection of mannan from *S. cerevisiae* (28). Here, we developed a modified mannan-induced psoriasis inflammation model, dependent mainly on innate immune cells, to study the effects of estrogen on psoriasis. This model showed a clear female preponderance in disease development and can be induced in different common mouse strains by epicutaneous applications of mannan, a natural route of antigen encounter in the skin.

At first, we compared psoriasis development between male and female mice and found an increased disease severity in females. Next, we investigated the role of endogenous and exogenous estrogen, as well as ER-β antagonist and agonist on experimental psoriasis. Interestingly, estrogen alone promoted the skin inflammation and increased mannan-induced psoriasis-like inflammation, which was mainly dependent on the IL-17 family of cytokines and keratinocyte proliferation, possibly by acting on ER-β.

## Materials and Methods

### Mice

Eight to twelve weeks old BALB/c, C57BL/6J, KM, DBA/1, C57BL/6NQ, ICR, and NIH male and female mice maintained in a pathogen-free animal house were purchased from Southern Medical University and Guangdong Medical Animal Experiment Center. All animal experiments were conducted in accordance with the guidelines of the National Institutes of Health (NIH Publication No. 8023) and approved by the ethics committee of Southern Medical University (l2018183). Mice were placed in cages, in a climate-controlled environment having 12-h light/dark cycles. All the procedures were approved and supervised by Southern Medical University Animal Care and Use Committee, Guangzhou, China.

### Mannan-Induced Psoriasiform Inflammation

The area of 2 cm x 3.5 cm was shaved at the back of mice after injection of pentobarbital sodium (80 mg/kg, Hechun Guangzhou, China), and 5 mg mannan (100 μl/day, Sigma-Aldrich, Missouri) mixed with incomplete Freund’s adjuvant (IFA, Sigma-Aldrich) in a ratio of 1:1 was applied on the back skin for 3 consecutive days and scored for 9 days. For the induction of disease relapse, at the end of initial psoriasis development (Day 9), a mixture of mannan and IFA was applied on the skin for another 3 days. Psoriasis area severity index (PASI) scores were given based on redness (0-4), scales (0-4), and thickness (0-4) with a total score of 12 by following the criteria: 0, none; 1, slight; 2, moderate; 3, severe and 4, very obvious signs. Increase in skin thickness was measured using an Ozaki digital caliper (Neill-Lavielle, Kentucky).

### Ovariectomy and Estradiol Treatment

Ovariectomy (OVX) or sham operation was performed on 5-6 weeks old BALB/c mice (n = 8-12/group). After 2 weeks, psoriasis-like skin inflammation was induced using mannan and monitored up to 9 days in the endogenous estrogen depletion experiments. For estrogen treatment, after evaluating different routes (**Supplementary Figure S1**) and concentration (**Supplementary Figure S1**), subcutaneous injection of 7.2 μg 17-β-estradiol (**Supplementary Figure S2**) was selected and used. In these experiments, two weeks after surgery, 17-β-estradiol (7.2 μg/day) or miglyol was given *s.c.* for 6 days and monitored for skin inflammation. Subsequently, mannan was applied from Days 7-9 with E2 or miglyol treatment and the disease development was monitored until Day 16. In ER-β antagonist treated group, PHTPP (1 mg/kg, MCE, New Jersey) was used in OVX mice for 9 days, and corn oil as well as E2 treated OVX mice were used as controls. DPN (12 mg/kg, MCE), an ER-β agonist was used in OVX mice for 9 days and corn oil was used as a control.

### Blood Circulation Measurements

BALB/c mice (n = 10/group) were anesthetized by injecting phenobarbital on Day 5 after mannan application and Laser Speckle Contrast Imager (LASCA analyzer, Zanda, Shanghai, China) was used to scan the back of the mouse with infrared light for 60 s. Pictures were recorded using the Laser Speckle Contrast Analysis. A specific time (25 s-35 s) was chosen for collecting blood circulation data from the skin.

### Histology and Immunohistochemistry

A group of mice was sacrificed on Day 6 and their skin samples were collected, fixed in 4% paraformaldehyde, embedded in paraffin, and stored at RT until used. Paraffin sections (8 μm) were cut using a paraffin slicer (Leica, Solmas, Germany) and stained with H&E (Beyotimes, Shanghai, China). Images of H&E staining were acquired using an eclipse upright optical microscope digital camera (Nikon, Tokyo, Japan). Two random positions from each mouse were selected to measure the epidermal thickness by Image J software (version 1.8.0, Maryland). Baker’s scores (29, 29) were used to analyze the pathological severity of the skin. CD11c positive cells were stained with biotin-rat anti-mouse CD11c antibody (1:100, Biolegend, California) for 1 h, followed by streptavidin-HRP antibody (1:800, Yeasen, Shanghai, China) incubation for 40 min. Sections were developed with DAB (Vector Laboratories, California) and counter-stained with hematoxylin (Phygene, Shanghai, China) before visualization under the microscope (Nikon). CD11c positive cells (30) were counted in 5 microscopic fields under 200X magnification and expressed as cells/field, and mean ± SEM.

### Immunofluorescence

For immunofluorescence staining, frozen skin sections were dehydrated, embedded, stained, and imaged using a confocal microscopy (A1HD25, Nikon). To evaluate the expression and location of ER-β, mouse skin tissues were incubated with the specific primary antibodies for rabbit anti-mouse ER-β (1:50, Abcam, Cambridge, UK), Alexa Fluor^®^ 488 conjugated Pan-Keratin (1:100, Abcam) and CD11c (1:100, BioLegend) overnight. Alexa Fluor^®^ 555 conjugated anti-rabbit IgG (1:800, Biyotimes) and Alexa Fluor^®^ 488 conjugated streptavidin (1:800, Biyotimes) were used as secondary reagents. Nuclear DNA was detected by DAPI (5 mg/ml, Biyotimes) for 5 min at room temperature. Confocal images were acquired using a Nikon Laser Confocal Microscope and analyzed using NIS Elements Viewer Imaging Software (Nikon).

### Flow Cytometry

Skin samples were separated into dermis and epidermis layers after digestion with dispase II (10 mg/ml, Solarbio, Beijing, China) for 2 h at 37°C. To obtain single cells, shredded dermal tissue was treated with collagenase II (3 mg/ml, Solarbio) and DNase I (5 mg/ml, Solarbio) at 37°C for 120 min. Single cells were also prepared from inguinal lymph nodes and spleen from mice by maceration. Surface staining was performed with fluorescent-labeled antibodies F4/80-PerCP-Cy 5.5, CD11c-PE, CD11b-APC, Ly6C/6G-FITC, CD45-PerCP-Cy 5.5, γδ T-PE (BD Biosciences, New Jersey) for 30 min at room temperature. FACS was performed using LSR II (BD Biosciences) and data were analyzed using Flow Jo version 7.0 (Tree Star, California). Gating strategy for different cell populations was given in **Supplementary Figure S3**.

### RNA Isolation and RT-PCR

Total RNA was extracted from the skin with Trizol reagent (Invitrogen, California) and dissolved in RNAse-free DEPC water (Phygene, Shanghai, China) before analysis. The mRNA was reverse-transcribed to cDNA with PrimeScript RT reagent Kit (ThermoFisher, Massachusetts). Whereas MiR-146a, miR-21 and miR-210 were specifically reverse transcribed with the Ribobio Bulge-Loop miRNA RT-qPCR kit (Ribobio, Guangzhou, China). Each RT-PCR was performed with SYBR Premix Ex Taq II (Takara biotech, Osaka, Japan) using a LightCycler 96 thermocycler (Roche, Basel, Switzerland). U6 or β-actin was used as a general quantitative control. Transcript levels were calculated relative to controls and the relative fold expression was calculated using the 2– ΔΔCt algorithm. The primers of different genes were given in **Supplementary Table S1**. Primers for miR-146a, miR-210, miR-21, and U6 were purchased from RiboBio.

### Statistical Analysis

The data were analyzed with GraphPad Prism 5 and are presented as mean ± SEM. Two-tailed unpaired Student’s t test was used for comparison between the two groups. One-way analysis of variance (ANOVA) with Bonferroni or Newman–Keuls correction was used for multiple comparisons. Probability values < 0.05 were considered significant for 95% confidence interval.

## Results

### Mannan-Induced Psoriasis-Like Skin Inflammation

**Figure 1:**
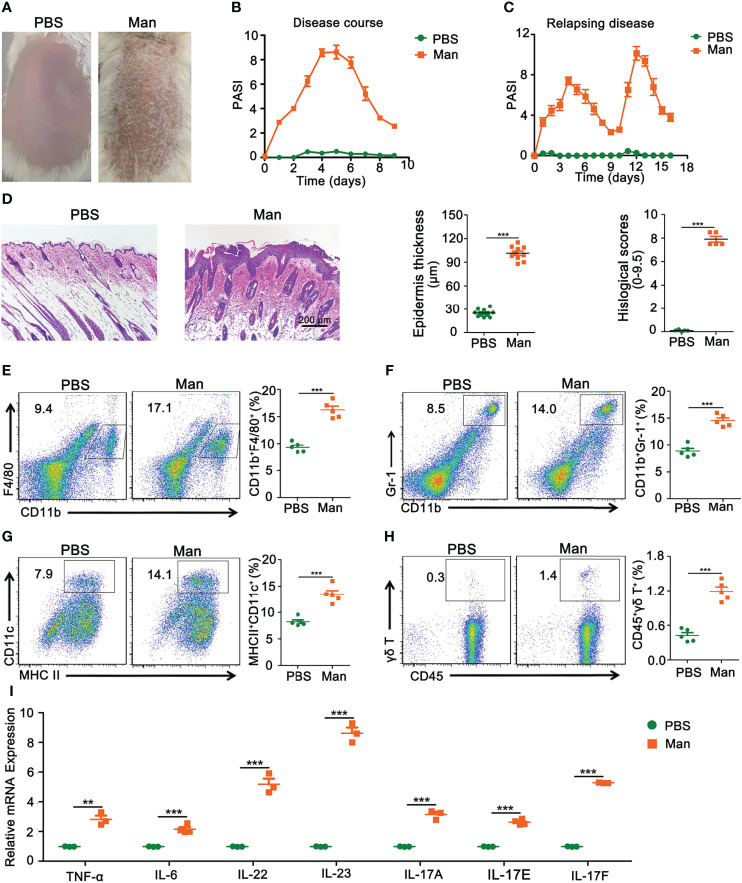
Characterization of mannan-induced skin inflammation. **(A)** Representative pictures of mouse skin after PBS or mannan application on Day 5 (n = 10/group). **(B)** Disease course after mannan or PBS application mixed with IFA in BALB/c mice on three consecutive days starting from Day 0 (n = 10/group); **(C)** relapsing mannan-induced skin inflammation (n = 10/group); **(D)** representative pictures of HE staining of psoriatic skin and statistical results of epidermal thickness with histological scores at peak of psoriasis (Scale bars: 200 μm) (n = 5/group). Representative pictures and statistical results on the expression of **(E)** macrophages (CD11b^+^F4/80^+^), **(F)** neutrophils (CD11b^+^Gr-1^+^), and **(G)** dendritic cells (MHCII^+^CD11c^+^) expression in spleen in mannan-induced skin inflammation on Day 5 (n = 5/group). **(H)** Representative pictures and percentage of γδT cells (CD45^+^γδT^+^) after PBS or mannan application on Day 5 in the draining lymph nodes (n = 5/group). **(I)** Expression of IL-23/IL-17 axis and its upstream cytokines (IL-6, TNF-α) (n = 3/group). Man, mannan. Statistical analyses were performed using an unpaired t test and n indicates number of mice in each group. The data represent mean ± SEM. **p < 0.01. ***p < 0.001.

We established a new psoriasis model in BALB/c mice with mannan from *Saccharomyces cerevisiae* (SC) cell wall. Plaque psoriasis was developed after a mannan mixture was applied epicutaneously for three consecutive days, and the disease symptoms reached the peak at Day 5 (**Figure 1A**). After initial mannan exposure, the peak of psoriasis was detected on Day 5, with a mean maximum psoriasis score of 9 and then the disease subsided (**Figure 1B**). Repeated mannan exposure at Day 9 caused a relapsing disease, with an increased severity (**Figure 1C**). On Day 5, inflamed skin had thickening of the outer layer of the skin (hyperkeratosis) as well as acanthosis with significantly increased histological scores (**Figure 1D**). There were fewer immune cells in the normal skin, while in the inflamed skin, cells were recruited from draining lymph nodes and spleen. A significant increase in the percentage of dendritic cells, neutrophils, and macrophages was found in the spleen after mannan stimulation (**Figures 1E–G**). A significantly higher expression of γδ T cells was found in the draining lymph nodes of inflamed mice (**Figure 1H**). The expression of TNF-α and IL-6 as well as IL-22, IL-23, and IL-17 family of cytokines (IL-17A, IL-17E, and IL-17F) was significantly increased in psoriasis skin lesions (**Figure 1I**).

### Females Developed A More Severe Psoriasis-Like Skin Inflammation Than Males

**Figure 2:**
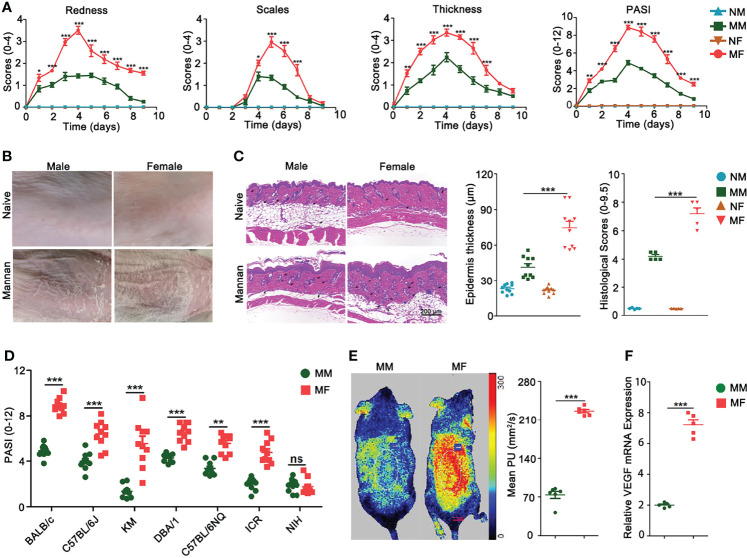
Female mice developed more severe psoriasis. **(A)** Comparison of redness, scales, thickness, and total psoriasis area and severity index (PASI) between BALB/c female and male mice after mannan application (n = 15/group). **(B)** Representative pictures of psoriasis and naive skin from female and male mice on Day 5 (n = 12/group). **(C)** Comparison of H&E-stained skin sections, epidermal thickness, and histological scores between naive and psoriatic BALB/c female and male mice (n = 5/group). Scale bar: 200 μm. Infiltration of immune cells is marked by black arrows. **(D)** Comparison of total PASI at the peak of psoriasis in BALB/c, C57BL/6J, KM, DBA/1, C57BL/6NQ, ICR, and NIH female and male mice on Day 5 (n = 10/group). **(E)** Blood flow (n = 6/group) and **(F)** VEGF gene expression (n = 5/group) in the psoriatic skin of female and male mice on Day 5 after mannan application. Mean level of blood flow as well as VEGF gene expression in the PBS treated female and male mice was taken as one. Each experiment was repeated twice. Statistical analyses were performed using an unpaired t test and n indicates number of mice. The data represent mean ± SEM. NM, naive male; MM, Mannan + Male; NF, Naive Female; MF, Mannan + Female. ns, not significant; *p < 0.05; **p < 0.01. ***p < 0.001.

In order to explore estrogen functions on mannan-induced skin inflammation, at first we detected differences in psoriasis scores between female and male mice. All the psoriasis area and severity index (PASI) parameters like redness, scales, and skin thickness were higher in female BALB/c mice (**Figure 2A**) as shown in the clinical pictures (**Figure 2B**) and H&E staining with an increased (twofold) epidermal thickness, infiltration of immune cells, and higher histological scores (**Figure 2C**). Similarly, females developed a more severe psoriasis in most of the tested mouse strains (BALB/c, C57BL/6J, C57BL/6NQ, KM, DBA/1, and ICR) (**Figure 2D**) except in NIH mouse strain. In addition, a higher level of blood flow in the skin (**Figure 2E**) and an increased vascular endothelial growth factor (VEGF) expression were observed in the diseased female mice (**Figure 2F**).

### Endogenous and Exogenous Estrogen Promoted Psoriasis

**Figure 3:**
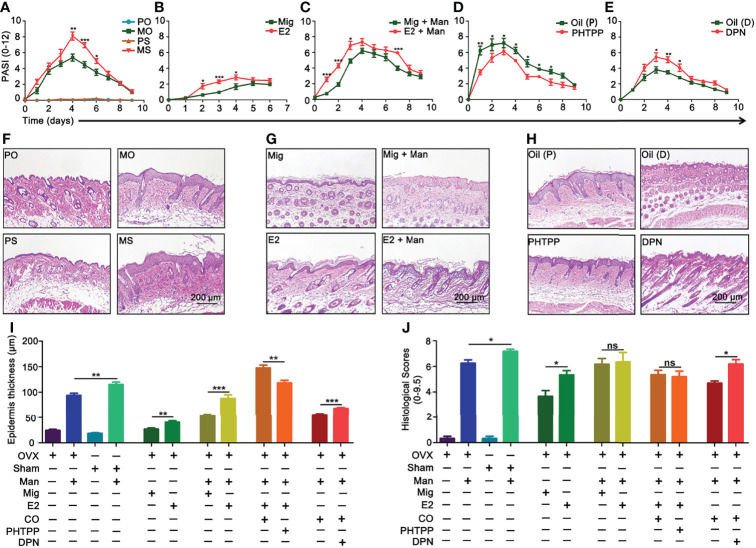
Endogenous and exogenous estrogen promoted psoriasis. Comparison of psoriasis scores (PASI): **(A)** Ovariectomized (OVX) and sham operated mice with or without mannan application (n = 10/group); **(B)** single 17-β-estradiol or miglyol alone treated mice without any mannan application (n = 10/group); **(C)** 17-β-estradiol or miglyol alone injected mice with mannan application (n = 10/group); **(D)** ER-β antagonist (PHTPP) or corn oil alone treated mice (n = 5/group), **(E)** ER-β agonist (DPN) or corn oil alone treated mice (n = 5/group). Representative pictures of H&E-stained skin samples at peak of psoriasis from **(F)** OVX and sham operated mice, **(G)** 17-β-estradiol treated OVX mice, without and with mannan stimulation, and **(H)** after PHTPP and DPN treatment. **(I, J)** Epidermis thickness (n = 5/group) and histological scores of skin sections (n = 3/group) at Day 5 were calculated from Figures **(F–H)**. Scale bar: 200 µm. Each experiment was repeated twice. Statistical analyses were performed using an unpaired t test and n indicates number of mice. The data represent mean ± SEM. Man, mannan; PO, PBS + OVX; MO, Man + OVX; PS, PBS + sham; MS, Man + Sham; Mig, miglyol; E2, 17-β-estradiol; Oil (P), E2 + Corn Oil + Man; PHTPP, E2 + PHTPP + Man; Oil (D), Corn Oil + Man; DPN, DPN + Man. The data represent mean ± SEM. ns, not significant. *p < 0.05; **p < 0.01. ***p < 0.001.

Depletion of endogenous estrogen by ovariectomy (OVX) had significantly decreased the PASI scores compared to sham operated mice (**Figure 3A**). Interestingly, a single injection of 17-β-estradiol alone led to psoriasis-like dermatitis (**Figure 3B**) and these symptoms were enhanced after mannan application (**Figure 3C**). Among the three known receptors of estradiol, a higher level of ER-β expression was found in the skin of female mice, which was also increased by endogenous and exogenous estrogen in the inflamed mice, but 17-β-estradiol alone had no such effect (**Supplementary Figure S2**). Next, we used ER-β antagonist PHTPP and agonist DPN to treat the inflamed mice. Psoriasis scores were decreased by PHTPP treatment (**Figure 3D**) but increased after DPN injection (**Figure 3E**) during the disease course. Histological evaluation of the inflamed skin samples showed endogenous estrogen enhanced epidermal thickness (**Figure 3F**) and an increased level of keratinocyte proliferation in the epidermis after 17-β-estradiol injection, whereas mild keratinization of epidermis was found with the injection of 17-β-estradiol alone (**Figure 3G**). On the other hand, epidermal thickness decreased after PHTPP treatment but increased after DPN injection, which correlated well with the PASI scores (**Figure 3H**). Quantification of stained skin sections confirmed the increase in epidermal thickness (**Figure 3I**) and histological scores induced by endogenous and exogeneous estrogen, treatment with 17-β-estradiol alone, or DPN (**Figure 3J**).

### Increased Dendritic Cell Numbers in the Inflammatory Skin AfterEstrogen Treatment

**Figure 4:**
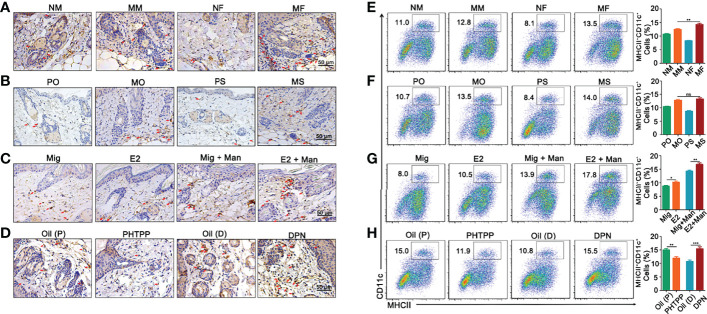
Dendritic cells from dermis and spleen contributed to estrogen promoted psoriasis. **(A)** Immunohistochemistry staining of CD11c^+^ cells in dermis (arrows mark representative cells) from female and male mice without or with mannan application (n = 5/group). Effect of **(B)** endogenous estrogen, **(C)** 17-β-estradiol, **(D)** PHTPP and DPN on CD11c^+^ cells in the dermis under normal or inflammatory conditions (n = 5/group). Scale bar: 50 µm. **(E)** Presence of MHCII^+^CD11c^+^ cells in the spleen from female and male mice (n = 5/group). Effect of **(F)** endogenous estrogen, **(G)** 17-β-estradiol, **(H)** ER-β antagonist or agonist on MHCII^+^CD11c^+^ cells (n = 5/group). Each experiment was repeated twice. Man, mannan; NM, Naive Male; MM, Man + Male; NF, Naive Female; MF, Man + Female; PO, PBS + OVX; MO, Man + OVX; PS, PBS + Sham; MS, Man + Sham; Mig, miglyol; E2, 17-β-estradiol; Oil (P), E2 + Corn oil + Man; PHTPP, E2 + PHTPP +Man; Oil (D), Corn oil + Man; DPN, DPN + Man. Statistical analyses were performed using an unpaired t test and n indicates number of mice used in each group. The data represent mean ± SEM. ns, not significant. *p < 0.05; **p < 0.01. ***p < 0.001.

Female mice had an increased expression of CD11c^+^ cells in the dermis (**Figure 4A**). Treatment with 17-β-estradiol and ER-β agonist but not endogenous estrogen had upregulated the percentage of CD11c^+^ cells, while ER-β antagonist decreased them in the inflamed skin (**Figures 4B–D**). Next, we confirmed our immunohistochemistry observations by immunofluorescence (**Supplemental Figure S4**). An increased number of MHCII^+^CD11c^+^ dendritic cells was found in the spleen from inflamed female mice (**Figure 4E**), with a negligible effect from endogenous estrogen (**Figure 4F**). Interestingly, treatment with17-β-estradiol increased the percentage of dendritic cells in the spleen both in naïve and inflamed mice (**Figure 4G**), while ER-β antagonist treatment significantly decreased their expression (**Figure 4H**).

### Estrogen Promoted Mannan Induced Skin Inflammation Was Dependent on ER-β Expression

**Figure 5:**
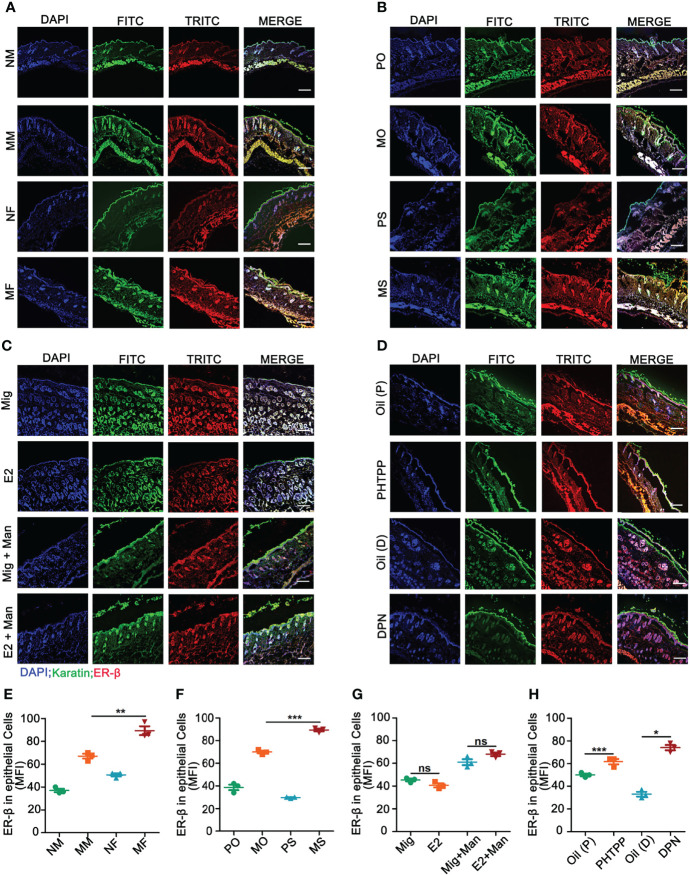
Estrogen increased mannan-induced skin inflammation promoted ER-β expression in keratinocytes. **(A)** Immunofluorescence staining of ER-β (red) and keratinocytes (green) in male and female mice with or without mannan application (n = 3/group); Effect of **(B)** endogenous estrogen, **(C)** 17-β-estradiol, **(D)** ER-β antagonist or agonist on ER-β expression in keratinocytes under normal and inflammatory conditions (n = 3/group). Scale bar: 200 µm. Nuclei were counterstained with DAPI (blue). **(E-H)** For quantification of immunofluorescence staining, mean fluorescence intensity of ER-β stained keratinocytes was calculated using Image J software. Representative pictures are shown. Man, mannan; NM, Naive Male; MM, Man + Male; NF, Naive Female; MF, Man + Female; PO, PBS + OVX; MO, Man + OVX; PS, PBS + Sham; MS, Man + Sham; Mig, miglyol; E2, 17-β-estradiol; Oil (P), E2 + Corn oil + Man; PHTPP, E2 + PHTPP +Man; Oil (D), Corn oil + Man; DPN, DPN + Man. Statistical analyses were performed using an unpaired t test and n indicates number of mice used in each group. The data represent mean ± SEM. ns, not significant. *p < 0.05; **p < 0.01. ***p < 0.001.

**Figure 6:**
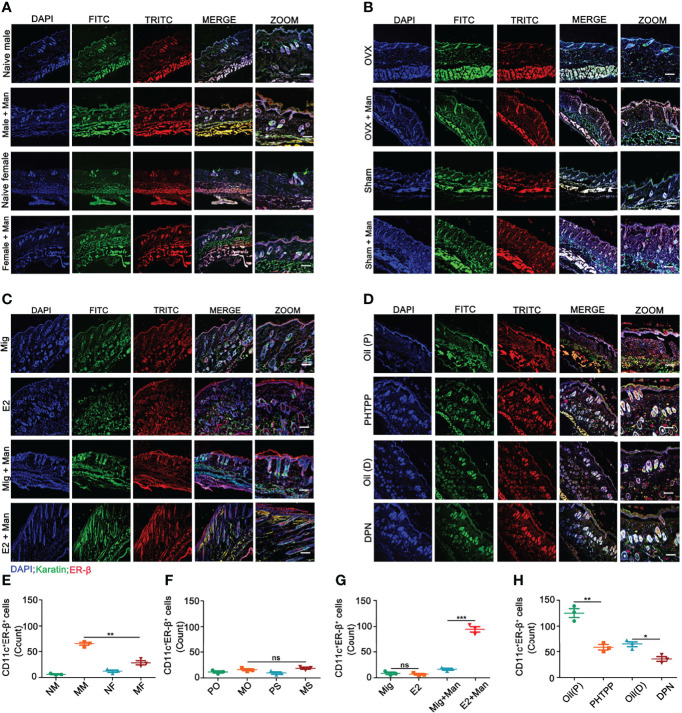
Estrogen increased ER-β expression in CD11c^+^ cells in the inflamed skin. **(A)** Immunofluorescence staining of ER-β (red) and CD11c (green) double positive cells in male and female mice under normal and inflammatory conditions (n = 3/group); effect of **(B)** endogenous estrogen, **(C)** 17-β-estradiol, **(D)** ER-β antagonist or agonist on the expression of ER-β and CD11c double positive cells (n = 3/group). **(E–H)** Quantification of CD11c^+^ER-β^+^ positive cells by immunofluorescence (n = 3/group). Scale bar: 200 µm. Nuclei were counterstained with DAPI (blue). Representative pictures are shown. Statistical analyses were performed using an unpaired t test. Man, Mannan; NM, Naive Male; MM, Man + Male; NF, Naive Female; MF, Man + Female; PO, PBS + OVX; MO, Man + OVX; PS, PBS + Sham; MS, Man + Sham; Mig, miglyol; E2, 17-β-estradiol; Oil (P), E2 + Corn oil + Man; PHTPP, E2 + PHTPP +Man; Oil (D), Corn oil + DPN; DPN, DPN + Man. n, number of mice. The data represent mean ± SEM. ns, not significant. *p < 0.05; **p < 0.01. ***p < 0.001.

Binding of estrogens to specific receptors (ER-α, ER-β, and GPR30) activate transcriptional processes and/or signaling cascades in the cells, which results in the regulation of gene expression and cellular functions. Among these receptors, expression of ER-β in the inflamed skin cells was more prominently affected by estrogen (**Supplementary Figure S4**). Next, we investigated ER-β expression in the skin cells by immunofluorescence. ER-β was mainly expressed in the epithelial cells, where an extensive keratinocyte proliferation was observed. Female mice had a higher-level ER-β expression in the keratinocytes under inflammatory conditions (**Figures 5A, E**). Interestingly, endogenous (**Figures 5B, F**) and ER-β antagonist (**Figures 5D, H**) but not treatment with 17-β-estradiol (**Figures 5C, G**) or ER-β agonist (**Figures 5D, H**) enhanced ER-β expression in the keratinocytes present in the inflamed skin. Subsequently, we detected ER-β expression in CD11c^+^ cells. Contrary to our expectations, more CD11c and ER-β double positive cells were detected in the male than female mice, though females had a significant increase in CD11c^+^ cells in the inflamed skin (**Figures 6A, E**). However, 17-β-estradiol injection (**Figures 6C, G**) but not endogenous estrogen (**Figures 6B, F**) had significantly increased the expression of these double positive cells, while both ER-β agonist and antagonist decreased their expression (**Figures 6D, H**). It is of interest to note that both RNA and protein levels of estrogen receptors are autoregulated (31), which might depend on the level and nature of their ligands present in the tissue, possibly regulated by epigenetic pathways.

### Estrogen Enhanced Mannan-Induced Skin Inflammation Was Promoted by γδ T Cells

**Figure 7:**
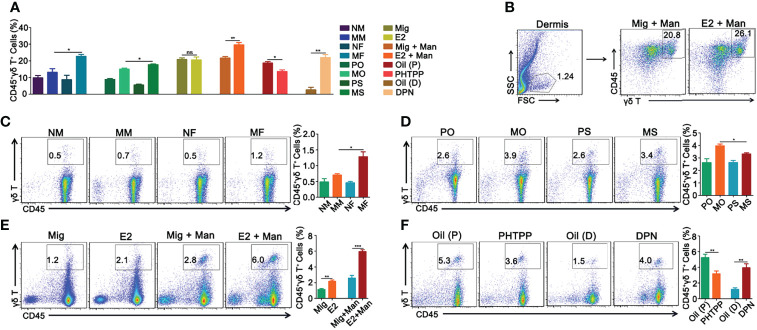
Estrogen enhanced mannan-induced skin inflammation was promoted by γδ T cells. **(A)** Percentage of CD45^+^γδ T^+^ cells in the dermis. **(B)** Representative pictures of CD45^+^γδ T^+^ cell expression after 17-β-estradiol treatment in MISI (n = 5/group). **(C–F)** γδ T^+^ cells in the draining lymph nodes from female and male mice, OVX and sham operated mice, 17-β-estradiol, miglyol, PHTPP or DPN treated mice with and without mannan application (n = 5/group). Each experiment was repeated twice. Man, mannan; NM, Naive Male; MM, Man + Male; NF, Naive Female; MF, Man + Female; PO, PBS + OVX; MO, Man + OVX; PS, PBS + Sham; MS, Man + Sham; Mig, miglyol; E2, 17-β-estradiol; Oil (P), E2 + Corn oil + Man; PHTPP, E2 + PHTPP + Man; Oil (D), Corn oil + Man; DPN, DPN + Man. Statistical analyses were performed using an unpaired t test and n indicates number of mice. The data represent mean ± SEM. ns, not significant. *p < 0.05; **p < 0.01. ***p < 0.001.

Females had significantly more γδ T cells than the male mice. Similarly, both 17-β-estradiol and ER-β agonist treated mice after mannan application had increased levels of γδ T cells, while ER-β antagonist treatment decreased their numbers in the dermis (**Figure 7A**). Representative pictures of increased percentage of γδ T cells in the dermis of inflamed mice after 17-β-estradiol treatment and a gating strategy used was shown in **Figure 7B**. In the draining lymph nodes, an increased percentage of γδ T cells was found in the female mice (**Figure 7C**) and in endogenous estrogen-depleted mice (**Figure 7D**). A single injection of 17-β-estradiol alone enhanced the percentage of γδ T cells in the draining lymph nodes, which was further increased by mannan application (**Figure 7E**). On the other hand, ER-β antagonist decreased the percentage of γδ T cells, while the agonist increased their expression in the draining lymph nodes (**Figure 7F**).

### Estrogen Promoted Psoriasis by Affecting Certain Genes, MicroRNAs,and Chemokines

**Figure 8:**
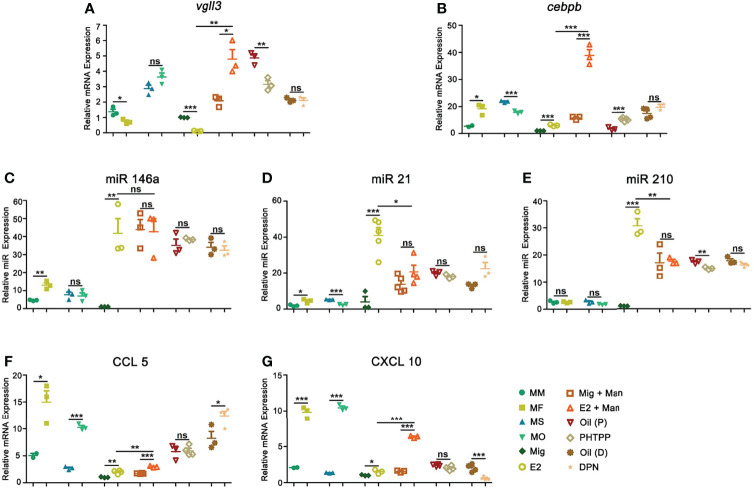
Estrogen increased psoriasis by affecting certain genes, microRNAs, and chemokines. Expression of **(A)** *Vgll3*, **(B)** *cebpb*, **(C)** miR146a, **(D)** miR21, **(E)** miR210, **(F)** CCL5, and **(G)** CXCL10 in male, female, OVX and sham operated, E2 or miglyol, PHTPP or DPN treated mice with and without mannan application (n = 5/group) in the skin. Each experiment was repeated twice. Man, mannan; MM, Man + Male; MF, Man + Female; MO, Man + OVX; MS, Man + Sham; Mig, miglyol; E2, 17-β-estradiol; Oil (P), E2 + Corn oil + Man; PHTPP, E2 + PHTPP + Man; Oil (D), Corn oil + Man; DPN, DPN + Man. Statistical analyses were performed using an unpaired t test. n, number of mice. The data represent mean ± SEM. ns, not significant. *p < 0.05; **p < 0.01. ***p < 0.001.

Although male mice had a higher level of *vgll3* expression, 17-β-estradiol supplement in OVX female mice after mannan application has significantly increased its expression, which was reduced by PHTPP (**Figure 8A**). On the other hand, a higher expression level of *cebpb* was found in the female mice after mannan application, while both endogenous and exogenous estrogen have significantly increased its expression (**Figure 8B**). Estrogen related microRNAs (miR146a, 21, and 210) promote inflammation in several autoimmune diseases, and here 17-β-estradiol alone or mannan application in female mice has significantly up-regulated the expression of these miRNAs (**Figures 8C–E**). Keratinocytes promote immune cell recruitment to psoriasis skin by secreting the chemokines, CXCL10 and CCL5 (32, 33), and we observed a higher level expression of these chemokine genes in the female mice. Similarly, 17-β-estradiol treatment in the naïve or inflamed mice has significantly up-regulated their expression, while ER-β agonist increased CCL5 but not CXCL10 expression (**Figures 8F, G**).

### Estrogen Promoted Psoriasis Inflammation Was Dependent onIL-23/IL-17 Axis

**Figure 9:**
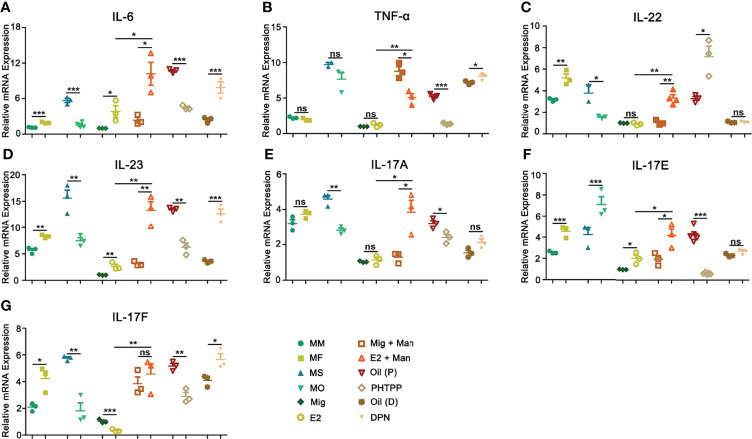
Estrogen promoted psoriasis symptoms were dependent on IL23/IL-17 axis. Expression of **(A)** IL-6, **(B)** TNF-α, **(C)** IL-22, **(D)** IL-23, **(E)** IL-17A, **(F)** IL-17E, and **(G)** IL-17F cytokines in male, female, OVX and sham operated, E2 or miglyol, PHTPP or DPN treated mice with and without mannan stimulation (n = 5/group). Man, mannan; MM, Man + Male; MF, Man + Female; MO, Man + OVX; MS, Man + Sham; Mig, miglyol; E2, 17-β-estradiol; Oil (P), E2 + Corn oil + Man; PHTPP, E2 + PHTPP +Man; Oil (D), Corn oil + Man; DPN, DPN + Man. Statistical analyses were performed using an unpaired t test and n indicates number of mice. The data represent mean ± SEM. ns, not significant. *p < 0.05; **p < 0.01. ***p < 0.001.

IL-6 secreted by macrophages promotes keratinocyte proliferation, which correlated with the expression of estrogen receptors (34). In this study, female mice had a higher-level expression of IL-6 and both endogenous and exogenous estrogen promoted its expression, which was confirmed by treatment with ER-β agonist or antagonist (**Figure 9A**). On the other hand, TNF-α expression did not show any clear pattern, though we observed its increase after DPN treatment, while treatment with PHTPP decreased its expression (**Figure 9B**). IL-22, produced in response to IL-6 and TNF-α, has a crucial function in the development of dermal inflammation and epidermal acanthosis(35). Here, both endogenous and exogenous estrogen have increased IL-22 expression (**Figure 9C**). In MISI, a significant increase in IL-23 expression was found in the female mice, which was further promoted by both endogenous and exogenous estrogen, and confirmed by treatment with ER-β agonist and antagonist (**Figure 9D**). An earlier report showed that Th17 family of cytokines (IL-17A and IL-17F) secreted by skin contained infiltrating γδ T cells and RORγt^+^ innate lymphocytes, which promoted the initiation of IMQ-induced psoriasis (36). In mannan-induced skin inflammation, an increased expression of the IL-17 family of cytokines (IL-17A, IL-17E, and IL-17F) was observed in the female mice. Similarly, endogenous and exogenous estrogen have also significantly enhanced their expression, while PHTTP treatment decreased it, though no effects were observed with DPN-treatment (**Figures 9E–G**).

## Discussion

The role of estrogen in psoriasis is often considered to be controversial and here we report a disease promoting role for estrogen using mannan-induced psoriasiform inflammation model. At first, we established a psoriasis model by epicutaneously applying 5 mg of mannan from *Saccharomyces cerevisiae* in BALB/c mice to explore the effects of estrogen in psoriasis. A significant increase in psoriasis scores, epidermal thickness, and blood circulation in the psoriatic skin of female mice was observed, possibly promoted by various genes and hormones. Both endogenous and exogenous estrogen have increased psoriasis scores, and ER-β expression was more pronounced in the skin, especially in keratinocytes. Next, we used ER-β antagonist and agonist to confirm the results. In addition, we observed significant effects of estrogen on keratinocytes, dendritic cells, and γδ T cells as well as cytokines and chemokines secreted by them under mannan-induced skin inflammation.

Sex bias was reported in many autoimmune diseases, such as systemic lupus erythematosus and rheumatoid arthritis, mainly related to X chromosomes and sex hormones, especially estrogen. Although there are no significant differences in psoriasis incidence between men and women, estrogen fluctuations during puberty, menstrual cycle, pregnancy, and menopause could have a profound effect on the severity of psoriasis (37). At the same time, estrogen was shown to affect resident and infiltrating cells present in the skin as well as chemokines/cytokines secreted by them during psoriasis development (38, 39). Therefore, we explored the effects of estrogen on psoriasis.

Mannan-induced skin abnormalities resembled different aspects of human plaque psoriasis with a macroscopic increase in redness and scales. Hyperkeratosis, acanthosis, innate inflammatory cell infiltration (dendritic cells, macrophages, neutrophils, and γδ T cells) and an increased expression of IL-23/IL-17 family of cytokines were also observed in this model. Interestingly, sex preponderance was more obvious. Females developed a more severe psoriasis in most of the tested mouse strains after mannan application. Similarly, after mannan stimulation, an increase in epidermal thickness, infiltration of immune cells, blood circulation, and the expression ER-β and VEGF were more prominent in female than male mice. Earlier, topical application of estrogen was shown to increase keratinocyte proliferation and epidermal thickness in the aged human skin (39), and promote endometrial angiogenesis by increasing VEGF expression in non-human primates (40), which could possibly suggest a disease promoting role for estrogen in females.

Next, we explored endogenous and exogenous estrogen effects on psoriasis. Both endogenous and exogenous estrogen have enhanced psoriasis scores after mannan application by increasing the thickness of the epithelial layer and infiltration of immune cells. Interestingly, a single subcutaneous injection of 17-β-estradiol alone induced psoriasis-like lesions with an increased expression of γδ T cells and miRNAs (miR146a, miR21, and miR210). Interestingly, promoter analysis of genes expressed in the skin of psoriatic patients showed an enrichment of the ER-β gene (41). However, ER-α activation has enhanced IL-23 secretion by DCs in IMQ-induced psoriasis (42), which contributed to disease severity. Here we showed a significant increase in ER-β but not ER-α/GPR30 in the skin samples, thereafter, we further confirmed the role of ER-β using its specific antagonist (PHTPP) and agonist (DPN). Treatment with PHTPP decreased PASI and epidermal thickness but without having any profound effect on histological scores. Whereas DPN increased psoriasis scores which was further confirmed by an increase in the epidermal thickness and histological scores.

In women with low estrogen levels, skin thickness was reduced approximately 1% per year after menopause (43). On the other hand, a topical estrogen administration in elderly males and females has significantly increased keratinocyte proliferation and epidermal thickness after 2 weeks (44). In addition, 17β-estradiol was earlier shown to promote keratinocyte proliferation by enhancing the expression of activated form of transcription factors like p-Akt and p-Erk (45). In this study, sex difference, endogenous and exogenous estrogen levels, ER-β antagonist as well as agonist treatment have significantly affected keratinocyte proliferation in the epithelial cells of psoriasis skin. Importantly, estrogen promoted ER-β expression most prominently in the keratinocytes under inflammatory conditions contributing to skin inflammation. These results are in agreement with an earlier study, in which estrogen was shown to promote keratinocyte proliferation *via* ER-β in a cutaneous wound healing mouse model (46).

Estrogen has significantly increased the expression of *Vgll3* and *cebpb* genes, which were earlier shown to regulate keratinocyte secreted IL-17 family of cytokines (20), as well as other chemokines and cytokines (47, 48) suggesting a possible involvement of these genes in estrogen promoted psoriasis. Several miRNAs (miR146a, miR21, and miR210) regulated by estrogen contribute to psoriasis development by regulating cytokine synthesis from keratinocytes and controlling their proliferation (49, 50). In this study, all these three miRNAs were significantly increased after a single injection of 17-β-estradiol suggesting their direct involvement in estrogen-induced proliferation of keratinocytes. On the other hand, 17-β-estradiol can control the proinflammatory signals/pathways of the immune system (51). Of note, estradiol and ER activity show clear dose- and context-dependent effects on immune signaling pathways and cell development (26).

IL-17A secretion from γδ T cells contributing to Ps-like inflammation was reported earlier by an intraperitoneal injection of mannan in ROS deficient BQ.Ncf1(m1j/m1j) mice (28, 52) and the direct relevance of γδ T cells was later demonstrated using γδ T knockout mice crossed to this mouse strain (53). Interestingly, 17-β-estradiol treatment in C57BL/6 OVX mice was shown to modulate γδ T cells, a major source of IL-17 (54) and in the imiquimod-induced psoriasis model, estrogen aggravated the disease by enhancing IL-23 secretion from dendritic cells (42). In this study, estrogen promoted mannan-induced skin inflammation by increasing the number of dendritic cells and γδT cells in the lymph nodes and skin, in addition to enhancing the expression of ER-β in keratinocytes and dendritic cells. Estrogen receptors are found in almost all cells of the immune system (26) and estrogen regulates several genes present in the cells of both innate and adaptive immune system (55). Interestingly, both ER-α and GPR30 are commonly associated with anti-inflammatory phenotypes, while ER-β, was shown to be associated with proinflammatory functions as well as an anti-inflammatory role. The differences in the effect of estrogen on cells could possibly be due to variations in receptor expression in cell types and prevailing different physiological states (56). However, the mechanisms behind sex differences in the expression of ERs in immune cells are not clear. In addition, how various concentrations of estrogens in men and women cause sex differences in ER expression in some cell types, but others are unknown but plausibly can be explained by epigenetic regulatory pathways (26).

Estrogen exhibits pro-inflammatory as well as anti-inflammatory effects through regulation of cytokine and chemokine synthesis, which depend on cell types, estrogen levels, and the inflammatory stimuli (57, 58). For example, chronic stimulation of murine macrophages with estrogen increased the production of pro-inflammatory cytokines (IL-1β, IL-6, TNF-α) (38) and it can also stimulate the activity of neutrophils (59). Earlier it was shown that estrogen can increase IL-17 production by splenocytes, which was further enhanced with an exposure to IL-23 (60). IL-17 in turn promotes inflammation by recruiting various inflammatory cells like neutrophils, monocytes, and macrophages to the site of inflammation and stimulating the target cells to secrete several inflammatory molecules. In this study, we observed a significant increase in the expression of IL-6, TNF-α, IL-22, IL-23, and IL-17 family of cytokines in the estrogen promoted mannan stimulated skin cells.

In conclusion, estrogen can affect psoriasis during various physiological periods of women, but few reports thus far focused on estrogen effects on psoriasis. Therefore, we established a mouse model in which we found a disease promoting role for estrogen. Both endogenous and exogenous estrogen increased mannan-induced psoriasis-like skin inflammation possibly by acting on ER-β. An increased *cebpb* expression, epidermal thickness, infiltration of dendritic cells, and γδ T cells as well as the expression of pro-inflammatory cytokines (IL-6, IL-23, and IL-17) and chemokines (CCL5 and CXCL10) could have contributed to this increase in psoriasis severity. These findings highlight the possible underlying mechanisms involved in estrogen promoted psoriasis in female mice. However, more studies are needed to address estrogen promoted cellular interactions and signaling pathways in the inflamed skin using individual cell populations.

## Data Availability Statement

The raw data supporting the conclusions of this article will be made available by the authors, without undue reservation.

## Ethics Statement

The animal study was reviewed and approved by Animal Care and Use Committee, Southern Medical University, Guangzhou, China (Approval no. l2018183).

## Author Contributions

HW did most of the experiments, analyzed data, prepared figures, and manuscript draft with contributions from KSN, who conceived the idea, designed experiments, supervised, interpreted the data, and modified the manuscript. LZ was involved in FACS experiments, and discussion for preparing figures and manuscript. JO contributed to FACS experiments. TW helped with the preparation of figures and analyzing results. YC modified the manuscript and helped with reagents. All the authors approved the content of this manuscript.

## Funding

This project was supported by “High-level talent introduction plan” project grants from Southern Medical University, Guangzhou, China (grant numbers C1034211, C1051004) given to KSN.

## Conflict of Interest

The authors declare that the research was conducted in the absence of any commercial or financial relationships that could be construed as a potential conflict of interest.

## Publisher’s Note

All claims expressed in this article are solely those of the authors and do not necessarily represent those of their affiliated organizations, or those of the publisher, the editors and the reviewers. Any product that may be evaluated in this article, or claim that may be made by its manufacturer, is not guaranteed or endorsed by the publisher.

## Supplementary Material

Supplementary Figure 1 Estradiol *via* subcutaneous route induced more severe disease. **(A)** Single subcutaneous injection of 17-β-estradiol induced more severe psoriasis-like inflammation than oral, intraperitoneal, and topical treatment (n = 5/group), **(B)** 17-β-estradiol promoted more severe mannan-induced skin inflammation through subcutaneous route. Mig, miglyol; E2, 17-β-estradiol; man, mannan (n = 5/group). Each experiment was repeated twice. Statistical analyses were performed using an unpaired t test. n indicates number of mice. The data represent mean ± SEM. *p < 0.05; **p < 0.01. ***p < 0.001.

Supplementary Figure 2 Effect of estradiol concentration on psoriasis. PASI scores from ovariectomized BALB/c mice treated with **(A)** 1.0 μg, **(B)** 3.6 μg, or **(C)** 7.2 μg of 17-β-estradiol (n = 10/group). Each experiment was repeated twice. Statistical analyses were performed using an unpaired t test. n indicates number of mice. The data represent mean ± SEM. *p < 0.05; **p < 0.01. ***p < 0.001.

Supplementary Figure 3 Gating strategy for γδ^+^T cells and innate immune cells. **(A)** Gating strategy for CD45^+^γδ^+^T from draining lymph nodes. Gating strategy for innate immune cells including **(B)** neutrophils (CD11b^+^Ly6C/6G^+^), **(C)** dendritic cells (MHCII^+^CD11c^+^), and **(D)** macrophages (CD11b^+^F4/80^+^) from the spleen. All the immune cells were stained and detected individually.

Supplementary Figure 4 Expression of estrogen receptors in the skin. Expression of **(A)** ER-α, **(B)** ER-β, and **(C)** G-protein coupled receptor 30 (GPR30) in naive, male, female, OVX, and sham operated as well as 17-β-estradiol treated mice with or without mannan application (n = 5/group). Man, mannan; MM, Man + Male; MF, Man + Female; MO, Man + OVX; MS, Man + Sham; Mig, miglyol; E2, 17-β-estradiol. Statistical analyses were performed using an unpaired t test. n indicates number of mice. The data represent mean ± SEM. *p < 0.05; **p < 0.01. ***p < 0.001.

Supplementary Figure 5 Immunofluorescence staining of CD11c^+^ dendritic cells in the skin. **(A)** Immunofluorescence staining of CD11c^+^ cells (green) in female and male mice in MISI (n = 3/group). Effect of **(B)** endogenous estrogen, **(C)** 17-β-estradiol, and **(D)** PHTPP or DPN on CD11c^+^ dendritic cells (n = 3/group). Scale bar: 200 µm. Nuclei were counterstained with DAPI (blue). For quantification of immunofluorescence staining, mean fluorescence intensity (MFI) of CD11c in the skin was calculated using Image J software in the above groups and the number of CD11c+ cells were counted manually. Man, mannan; NM, Naive Male; MM, Man + Male; NF, Naive Female; MF, Man + Female; PO, PBS + OVX; MO, Man + OVX; PS, PBS + Sham; MS, Man + Sham; Mig, miglyol; E2, 17-β-estradiol; Oil (P), E2 + Corn oil + Man; PHTPP, E2 + PHTPP +Man; Oil (D), Corn oil + Man; DPN, DPN + Man. Statistical analyses were performed using an unpaired t test and n indicates the number of mice used in each group. The data represent mean ± SEM. ns, not significant. *p < 0.05; **p < 0.01. ***p < 0.001.

## Abbreviations

CFA, complete Freund’s adjuvant; DCs, dendritic cells; DPN, diarylpropionitrile; E2, 17-b-estradiol; ER-α, estrogen receptor α; ER-β, estrogen receptor β; IFA, incomplete Freund’s adjuvant; IMQ, Imiquimod; KCs, keratinocytes; MISI, mannan-induced skin inflammation; PASI, psoriasis area and severity index; PHTPP, 4-(2-phenyl-5,7-bis[trifluoromethyl]pyrazolo[1,5-a] pyrimidin-3-yl) phenol.
