# Supplementary figures and images for: Estrogen Acts Through Estrogen Receptor-β to Promote Mannan-Induced Psoriasis-Like Skin Inflammation
Source: Front Immunol. 2022 May 19;13:818173. doi: 10.3389/fimmu.2022.818173 (PMC9160234; doi:10.3389/fimmu.2022.818173)

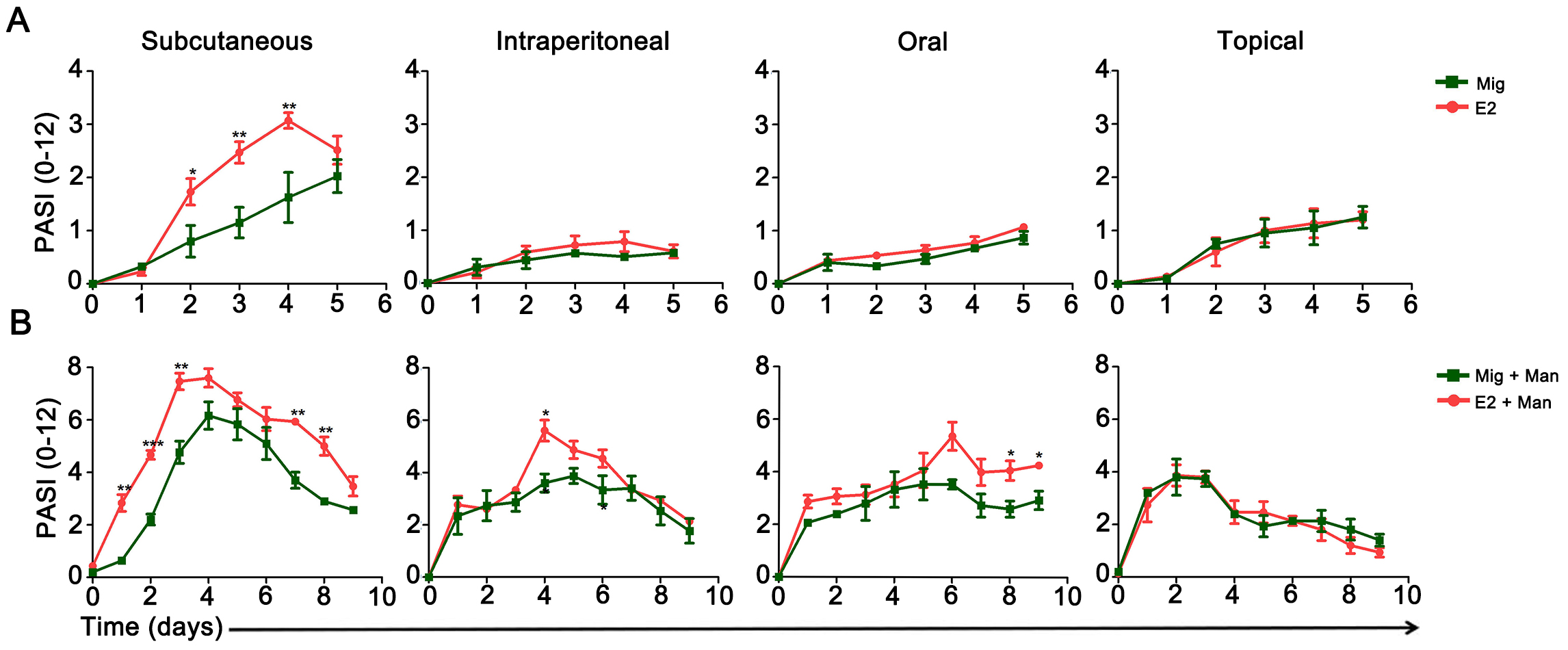

Supplement: Supplementary Figure 1 — Estradiol via subcutaneous route induced more severe disease. (A) Single subcutaneous injection of 17-β-estradiol induced more severe psoriasis-like inflammation than oral, intraperitoneal, and topical treatment (n = 5/group), (B) 17-β-estradiol promoted more severe mannan-induced skin inflammation through subcutaneous route. Mig, miglyol; E2, 17-β-estradiol; man, mannan (n = 5/group). Each experiment was repeated twice. Statistical analyses were performed using an unpaired t test. n indicates number of mice. The data represent mean ± SEM. *p < 0.05; **p < 0.01. ***p < 0.001. [file Image_1.jpeg]

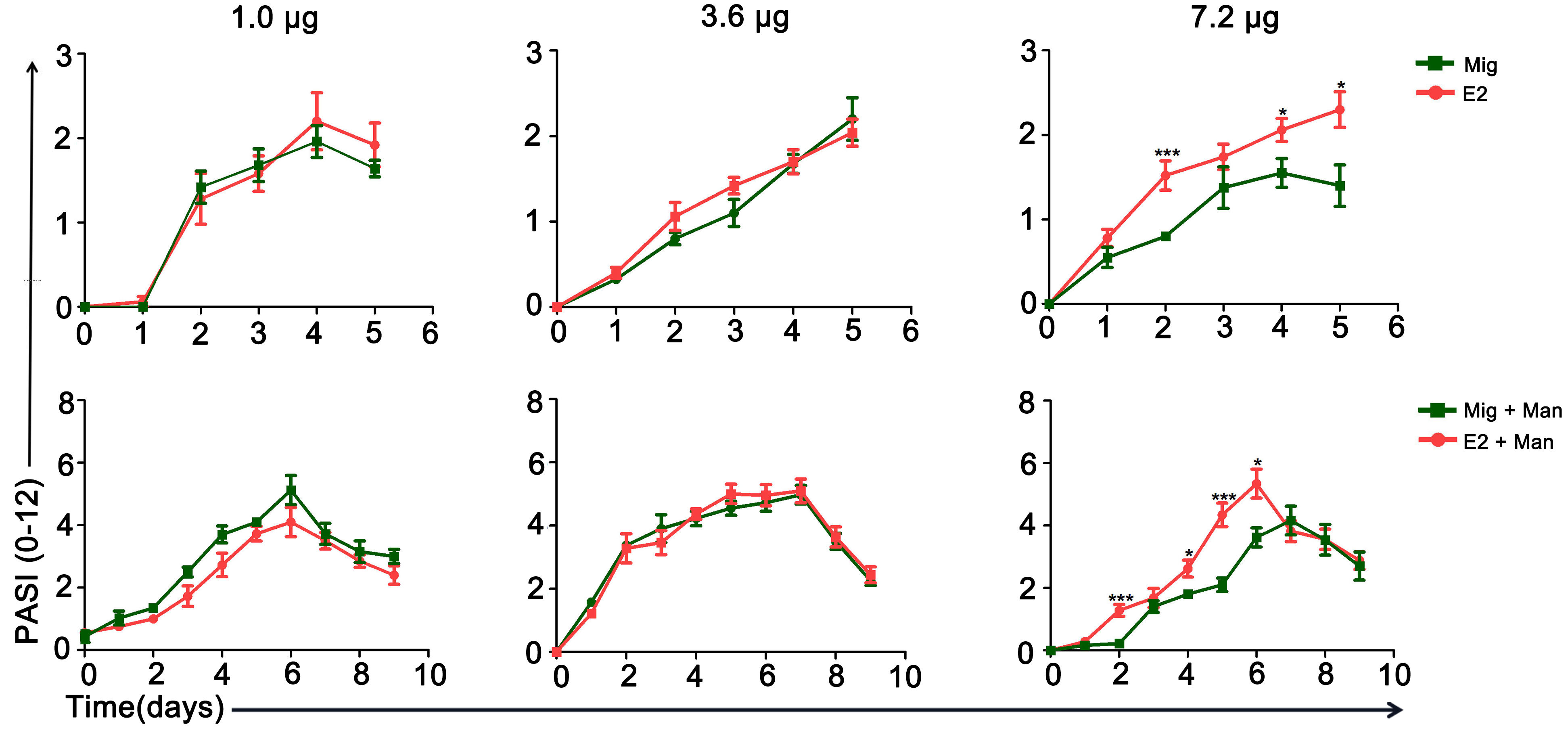

Supplement: Supplementary Figure 2 — Effect of estradiol concentration on psoriasis. PASI scores from ovariectomized BALB/c mice treated with (A) 1.0 μg, (B) 3.6 μg, or (C) 7.2 μg of 17-β-estradiol (n = 10/group). Each experiment was repeated twice. Statistical analyses were performed using an unpaired t test. n indicates number of mice. The data represent mean ± SEM. *p < 0.05; **p < 0.01. ***p < 0.001. [file Image_2.jpeg]

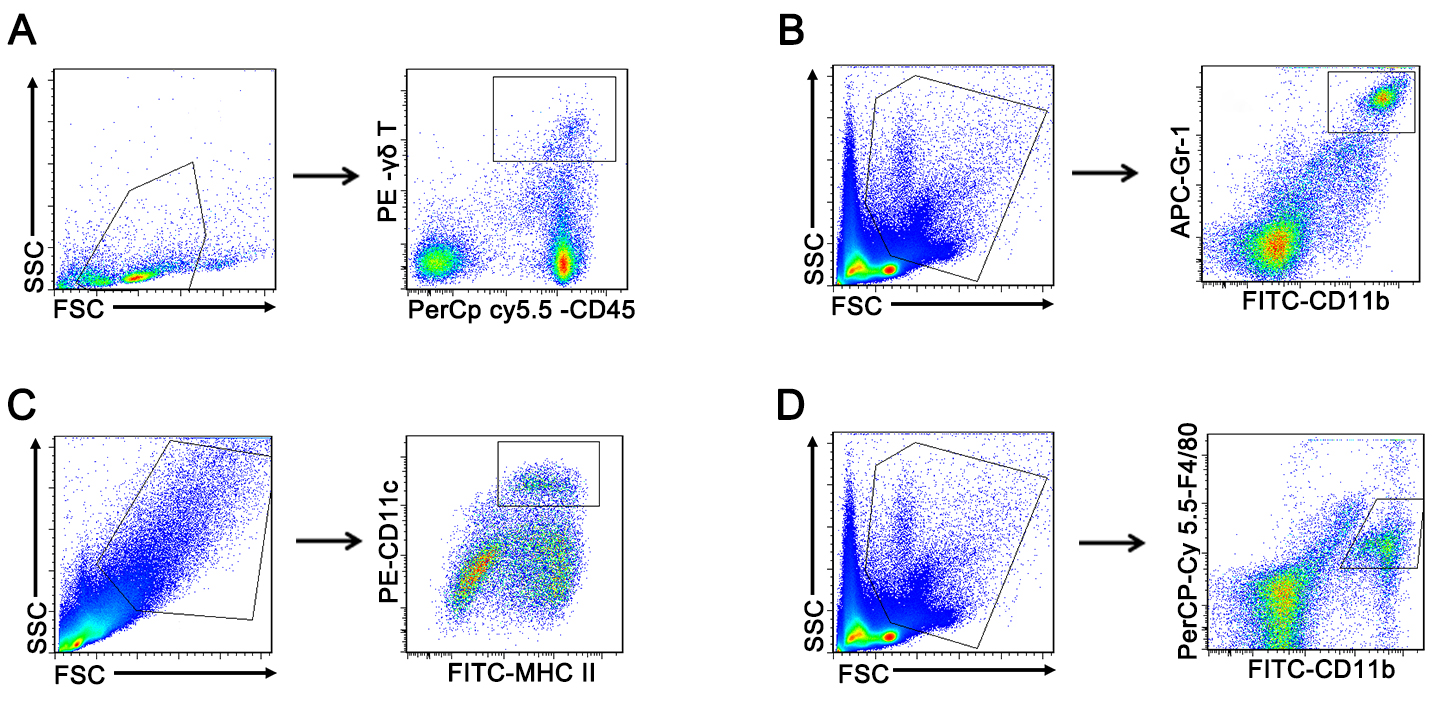

Supplement: Supplementary Figure 3 — Gating strategy for γδ+T cells and innate immune cells. (A) Gating strategy for CD45+γδ+T from draining lymph nodes. Gating strategy for innate immune cells including (B) neutrophils (CD11b+Ly6C/6G+), (C) dendritic cells (MHCII+CD11c+), and (D) macrophages (CD11b+F4/80+) from the spleen. All the immune cells were stained and detected individually. [file Image_3.jpeg]

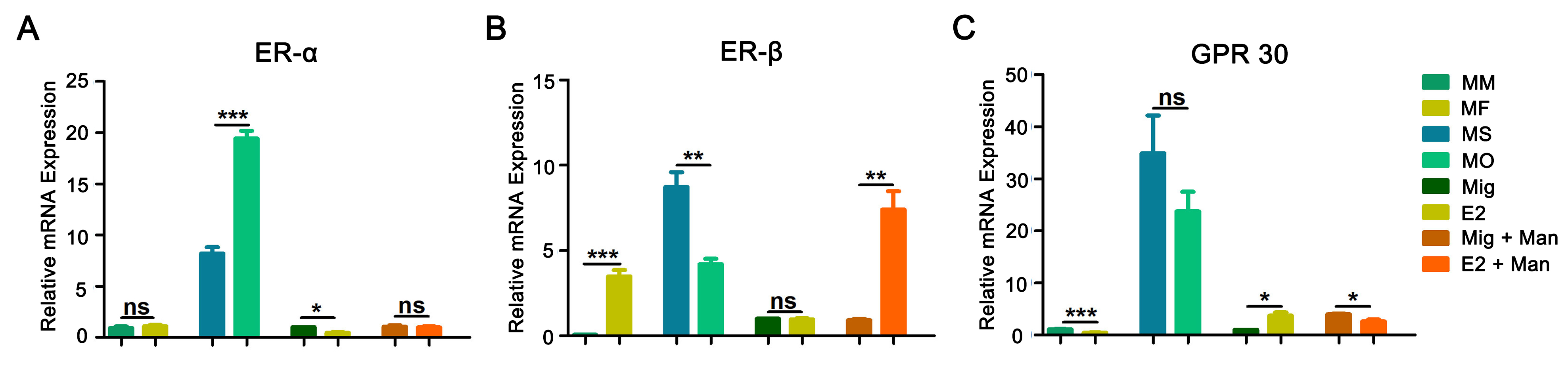

Supplement: Supplementary Figure 4 — Expression of estrogen receptors in the skin. Expression of (A) ER-α, (B) ER-β, and (C) G-protein coupled receptor 30 (GPR30) in naive, male, female, OVX, and sham operated as well as 17-β-estradiol treated mice with or without mannan application (n = 5/group). Man, mannan; MM, Man + Male; MF, Man + Female; MO, Man + OVX; MS, Man + Sham; Mig, miglyol; E2, 17-β-estradiol. Statistical analyses were performed using an unpaired t test. n indicates number of mice. The data represent mean ± SEM. *p < 0.05; **p < 0.01. ***p < 0.001. [file Image_4.jpeg]

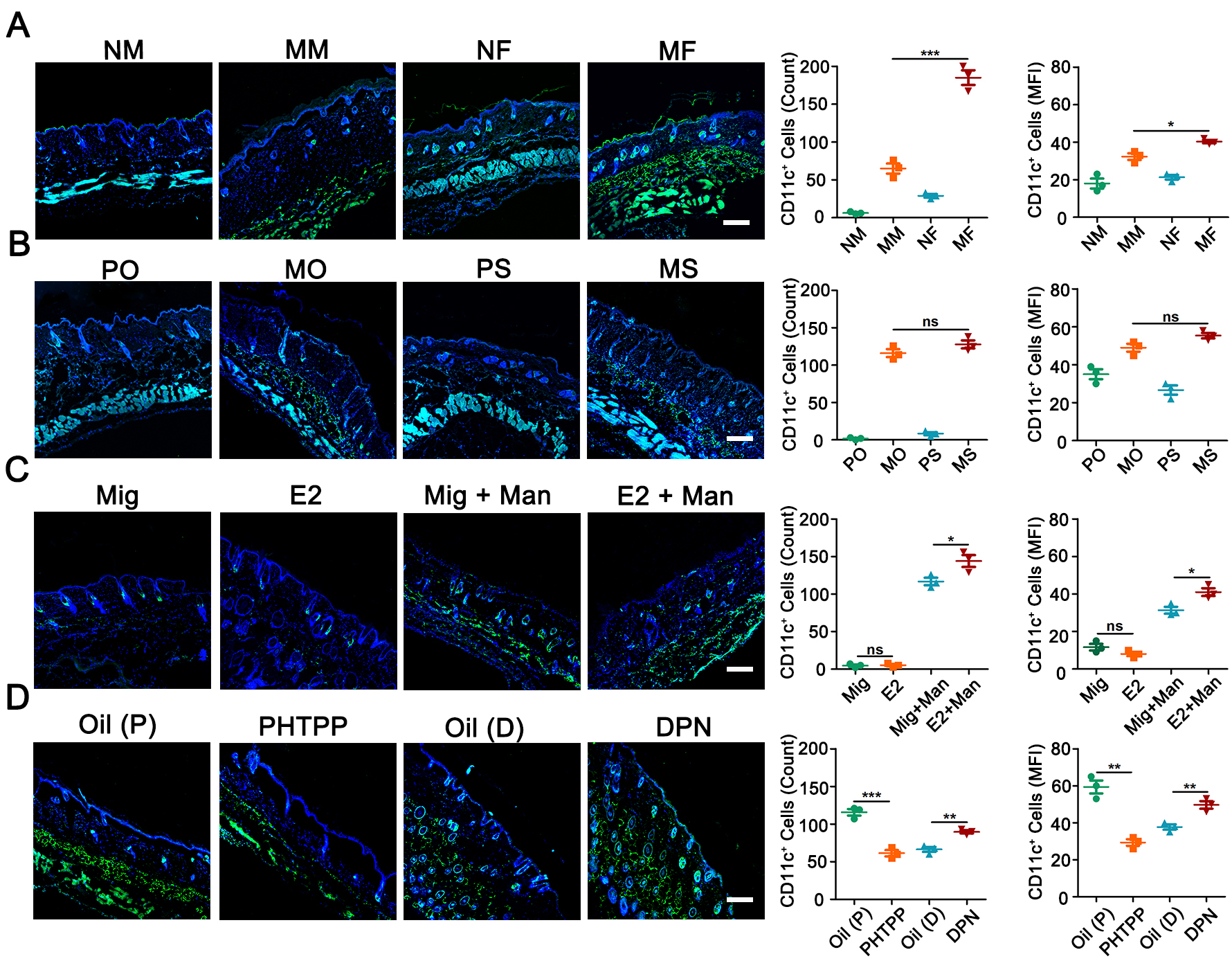

Supplement: Supplementary Figure 5 — Immunofluorescence staining of CD11c+ dendritic cells in the skin. (A) Immunofluorescence staining of CD11c+ cells (green) in female and male mice in MISI (n = 3/group). Effect of (B) endogenous estrogen, (C) 17-β-estradiol, and (D) PHTPP or DPN on CD11c+ dendritic cells (n = 3/group). Scale bar: 200 µm. Nuclei were counterstained with DAPI (blue). For quantification of immunofluorescence staining, mean fluorescence intensity (MFI) of CD11c in the skin was calculated using Image J software in the above groups and the number of CD11c+ cells were counted manually. Man, mannan; NM, Naive Male; MM, Man + Male; NF, Naive Female; MF, Man + Female; PO, PBS + OVX; MO, Man + OVX; PS, PBS + Sham; MS, Man + Sham; Mig, miglyol; E2, 17-β-estradiol; Oil (P), E2 + Corn oil + Man; PHTPP, E2 + PHTPP +Man; Oil (D), Corn oil + Man; DPN, DPN + Man. Statistical analyses were performed using an unpaired t test and n indicates the number of mice used in each group. The data represent mean ± SEM. ns, not significant. *p < 0.05; **p < 0.01. ***p < 0.001. [file Image_5.jpeg]
