# Supplementary material for: Estrogen Acts Through Estrogen Receptor-β to Promote Mannan-Induced Psoriasis-Like Skin Inflammation
Source: Front Immunol. 2022 May 19;13:818173. doi: 10.3389/fimmu.2022.818173 (PMC9160234; doi:10.3389/fimmu.2022.818173)
Supplement: Supplementary file 6 [file Table_1.pdf]

Table 1

Target gene	Direction	Primer sequence (5’ to 3’)
CEPBD	Forward	CCAAGCCGAGCAAGAAGC
	Reverse	CAGGGCGAACGGAAACC
VGLL3	Forward	AGGGAGACATTGGGTCAGTAG
	Reverse	ATTACTCCGCTGGCTCGAAAG
CCL 5	Forward	GTGTGCCAACCCAGAGAAGAAGT
	Reverse	AGCAAGCAATGACAGGGAAGCT
CXCL 10	Forward	CCTCTCTCCATCACTCCCCTTTA
	Reverse	TTGCTTCGGCAGTTACTTTTGTC
β-actin	Forward	ACCGTGAAAAGATGACCCAG
	Reverse	GTACGACCAGAGGCATACAG
IL-6	Forward	GAGAAAAGAGTTGTGCAATGGC
	Reverse	CCAGTTTGGTAGCATCCATCAT
IL-17A	Forward	CCCCTAAGAAACCCCCACG
	Reverse	TAAAGTCCACAGAAAAACAAACACG
IL-17E	Forward	ACAGGGACTTGAATCGGGTC
	Reverse	TGGTAAAGTGGGACGGAGTTG
IL-17F	Forward	GTCAGGAAGACAGCACCA
	Reverse	AGCCAACTTTTAGGAGCA
IL-22	Forward	CATGCAGGAGGTGGTACCTT

	Reverse	CAGACGCAAGCATTTCTCAG
IL-23-P19	Forward	AGCAACTTCACACCTCCCTAC
	Reverse	ACTGCTGACTAGAACTCAGGC
TNF-α	Forward	ACGCTCTTCTGTCTACTGAACT
	Reverse	ATCTGAGTGTGAGGGTCTGG
ER-α	Forward	CCTCCCGCCTTCTACAGGT
	Reverse	CACACGGCACAGTAGCGAG
ER-β	Forward	CTGTGCCTCTTCTCACAAGGA
	Reverse	TGCTCCAAGGGTAGGATGGAC
GPR 30	Forward	ATGGATGCGACTACTCCAGC
	Reverse	AAGAGGGCAATCACGTACTGC
U6	serial number	MQPS0000002-1-100
MiR-146a-5p	serial number	MIMAT0000158
MiR-21a-5p	serial number	MIMAT0000530
MiR-210-5p	serial number	MIMAT0000658
